# Indirect routes to reproductive success

**DOI:** 10.7554/eLife.00240

**Published:** 2012-10-15

**Authors:** John Pickett

**Affiliations:** Rothamsted Research, Harpenden, United Kingdomjohn.pickett@rothamsted.ac.uk

**Keywords:** Nicotiana attenuata, herbivory-induced plant volatile, plant-predator interaction, green leaf volatile, trypsin protease inhibitor, indirect defense

## Abstract

By comparing wild-type and transgenic tobacco plants in a natural ecosystem, researchers have confirmed that the indirect defence mechanisms employed by plants to fend off herbivorous insects can increase Darwinian fitness.

**Related research article** Schuman M, Barthel K, Baldwin I. 2012. Herbivory-induced volatiles function as defences increasing fitness of the native plant *Nicotiana attenuata* in nature. *eLife* **1**: e00007. doi: 10.7554/eLife.00007

**Image** Performing an assay on a tobacco plant

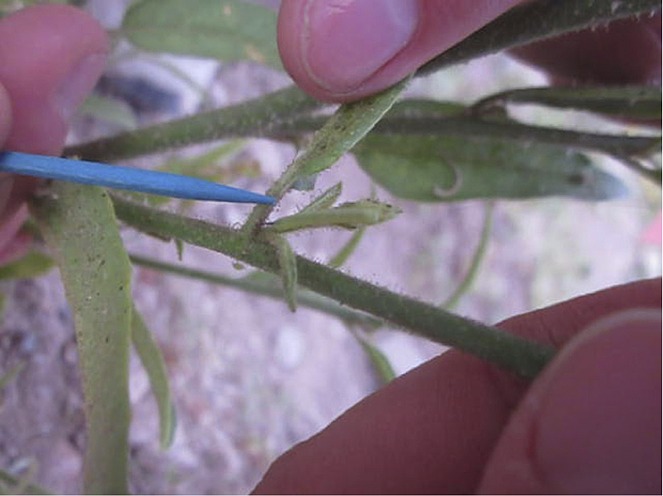

We have known for at least 30 years that the volatile compounds released by a plant when it is under attack by a herbivorous insect can enhance foraging behaviour by other insects that are either predators or parasites of the herbivore (Price et al., 1980). However, we have not had conclusive evidence that releasing these volatile compounds increased the fitness of the plant in the Darwinian sense of increasing reproductive success. Now, writing in *eLife*, Meredith Schuman, Kathleen Barthel and Ian Baldwin report such evidence from a two-year field trial on *Nicotiana attenuata*, a wild tobacco plant (Schuman et al., 2012). The work could pave the way to new approaches to pest management in agriculture that could reduce the need for pesticides, which is welcome at a time when new approaches to sustainable intensification of food production are being planned on a global scale (Royal Society, 2009).

Plants employ both direct and indirect defence mechanisms against herbivorous insects. Direct methods include the growth of prickles to fend off or injure the insects, and the release of chemicals that are toxic to herbivores or disrupt their digestive systems. However, when a herbivorous insect resists these direct defences, the plant may employ indirect mechanisms in response to both the physical damage caused by the insects and to the chemicals that they release. It is thought that the insects release these chemicals, which are called elicitors, to help them digest the plant (Turlings et al., 1990; Turlings et al., 2000). However, these chemicals also cause the plant to retaliate by releasing volatile compounds, and these volatiles attract parasitic wasps and other insects that attack the herbivore. These volatiles are also produced by the plant when the insect lays eggs on it, which means that the plant can respond to elicitors even when the insect does not cause any physical damage (Tamiru et al., 2011).

Schuman and co-workers, who are based at the Max Planck Institute for Chemical Ecology in Jena, performed a two-year field trial with wild tobacco plants in the Great Basin Desert in the south west of Utah. The herbivore was a type of caterpillar called *Manduca sexta*, which is also known as tobacco hornworm. When a tobacco plant is attacked by one of these caterpillars, the damage caused to the plant induces the release of green-leaf volatile (GLV) compounds in the form of six-carbon aldehydes, alcohols, and their esters. These molecules can have two different forms, called (*Z*)-3-GLVs and (*E*)-2-GLVs, which have the same chemical formulae but different arrangements of double bonds. Plants usually emit mostly (*Z*)-3-GLVs, but the elicitor changes some of the these into (*E*)-2-GLVs, which are more biologically active. These volatile signals attract various species of *Geocoris*, big-eyed bugs that prey on the caterpilars and therefore reduce damage to the tobacco plants (Figure 1; Allmann and Baldwin, 2010).

**Figure 1.**
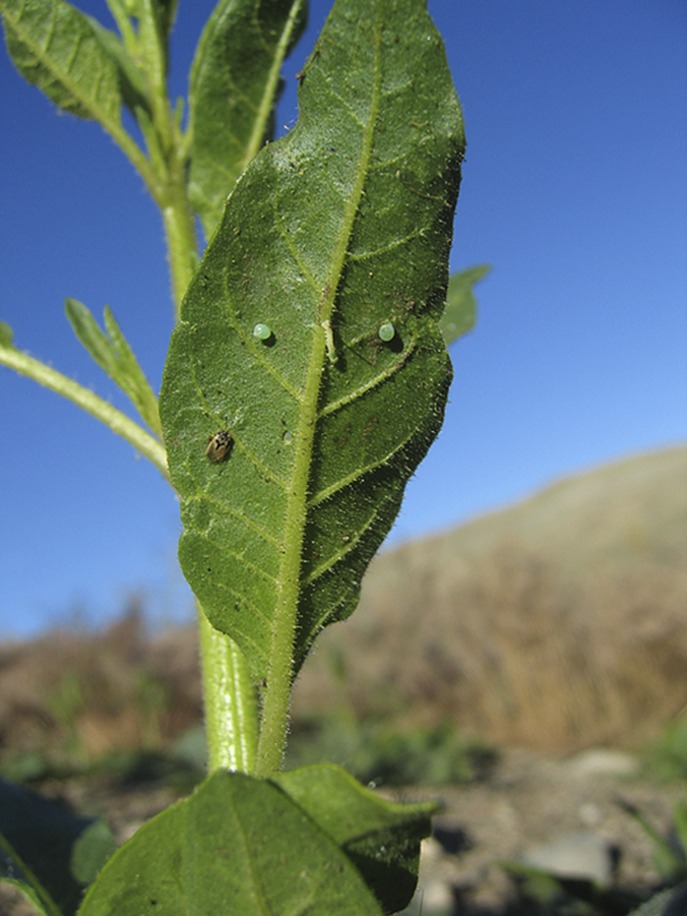
The wild tobacco plant *N. attenuata* relies on both direct and indirect mechanisms to defend it against *M. sexta* caterpillars. Indirect defence involves the release of volatile chemicals that attract *Geocoris* bugs that prey on the caterpillars. This photograph shows a *Geocoris* bug (bottom left) about to attack a caterpillar and two of its larvae.

PHOTOGRAPH: D KESSLER, MAX PLANCK INSTITUTE FOR CHEMICAL ECOLOGY

The work of Schuman and co-workers is driven by the hypothesis that an indirect defence mechanism—in this case, the release of volatiles to attract predators and parasites of the insects, a process called volatile herbivory-induced signalling—would increase plant reproduction (and, therefore, Darwinian fitness) by increasing predation of herbivores, whereas direct defence alone would not. To test this hypothesis, the Max Planck team compared wild-type *N. attenuata* and transgenic plants in which the biosynthetic pathway induced by the elicitors was knocked out by genetic modification. This means that the transgenic plants employ only direct defence (by releasing protease inhibitor proteins that disrupt enzymes in the digestive system of the herbivores), whereas the wild-type plants employ both direct defence and indirect defence (in the form of herbivory-induced signalling).

The results were unequivocal: herbivore-induced signalling caused a twofold increase in predation of the caterpillars, which was directly associated with a twofold increase in bud and flower production in the plants. The direct defence mechanism did not lead to such a large increase. I am sure Darwin himself would have been delighted. Alas, it was not possible to increase Darwin’s delight further by investigating the actual reproduction of the plants because the US Animal and Plant Health Inspection Service (APHIS) does not allow genetically-modified plants to disperse ripe seeds (and the measures needed to prevent the dispersal of seeds would have interfered with the field trial protocols that are essential in such experiments). However, the APHIS is to be complimented for allowing the trial to go ahead, and for showing how it is possible to improve our understanding of plant ecology by deploying genetically modified plants in the natural environment. This would not have been allowed in many European countries.

From the point of view of long-term practical applications in agriculture, it is more important to perform these experiments in a natural ecosystem, as Baldwin and co-workers have done, than it is to study these processes in crop plants. However, we know that the type of indirect defence mechanism demonstrated by wild tobacco plants can be depleted in crops that have been cultivated for use in intensive farming (Tamiru et al., 2011) and that it will be necessary to capture genetics from wild plants, including the ancestors of modern crops, by advanced breeding approaches (such as alien introgression), and also by genetic modification. What we have here is a dramatic demonstration of what nature can achieve, and how it should be possible to develop new approaches to agriculture in which pesticides are replaced by seed-delivered traits. This approach could also reduce or eliminate the need for energy-intensive processes in agriculture such as the manufacture and spreading of fertilizer: this would also reduce emissions of greenhouse gases.

We should also take this work as a call to expand our search for further opportunities to learn how nature deals with plant attack and to devise new ways of introducing these traits into crop protection. And where this relates to the genetic modification of plants, governments around the world need to support their registration agencies in devising routes by which these technologies can be used, supported by risk assessments that take into account the advantages of the new approaches and, in particular, the need to promote sustainability in food production, rather than mostly concentrating on the identification of potential hazards.
